# The Metric System

**Published:** 1879-07

**Authors:** 


THE METRIC SYSTEM.—Dr. Edward Wigglesworth, of Boston, is warming up considerably on the metric system. He is beginning to abuse our people in good set terms about their hesitation in the matter. Hear the way he closes his last manifesto :

" We need a benevolent despot who would compel the use of the metric system here after a fixed day. After a week no one would have any more trouble; after a month people would wonder how they could ever have used anything else, the labor of learning is so slight, and the gain so immense.

" All the poor peasants of Europe, the lowest classes of 'effete despotisms,' etc., have been able to adopt it at once, and yet Americans, self-ruling, are really too lazy, while merely claiming to be too stupid so to do. Shame on a country which 'to party gives up what was meant for mankind.' "—*Med. and Surg. Reporter.*

